# Influence of physical strain at high altitude on the quality of cardiopulmonary resuscitation

**DOI:** 10.1186/s13049-020-0717-0

**Published:** 2020-03-06

**Authors:** Alexander Egger, Maximilian Niederer, Katharina Tscherny, Josef Burger, Verena Fuhrmann, Calvin Kienbacher, Dominik Roth, Wolfgang Schreiber, Harald Herkner

**Affiliations:** 1 Mountain Rescue Service Austria, Schelleingasse 26/2/2, 1040 Wien, Austria; 2 Department of Anaesthesiology and Intensive Care Medicine, Hospital Scheibbs, Eisenwurzenstraße 26, 3270 Scheibbs, Austria; 3 Department of Emergency Medicine, Medical University of Vienna, Spitalgasse 23, 1090 Wien, Austria; 4 Department of Paediatrics, Hospital Lienz, Emanuel von Hibler-Straße 5 A, 9900 Lienz, Austria

**Keywords:** Cardiopulmonary resuscitation (CPR), Manikin, Mountain medicine, Out- of-hospital CPR

## Abstract

**Background:**

High quality cardiopulmonary resuscitation is a key factor in survival with good overall quality of life after out-of-hospital cardiac arrest. Current evidence is predominantly based on studies conducted at low altitude, and do not take into account the special circumstances of alpine rescue missions. We therefore aimed to investigate the influence of physical strain at high altitude on the quality of cardiopulmonary resuscitation.

**Methods:**

Alpine field study. Twenty experienced mountaineers of the Austrian Mountain Rescue Service trained in Basic Life Support (BLS) performed BLS on a manikin in groups of two for 16 min. The scenario was executed at baseline altitude and immediately after a quick ascent over an altitude difference of 1200 m at 3454 m above sea level. The sequence of scenarios was randomised for a cross over analysis.

Quality of CPR and exhaustion of participants (vital signs, Borg-Scale, Nine hole peg test) were measured and compared between high altitude and baseline using random-effects linear regression models.

**Results:**

The primary outcome of chest compression depth significantly decreased at high altitude compared to baseline by 1 cm (95% CI 0.5 to 1.3 cm, *p* < 0.01). There was a significant reduction in the proportion of chest compressions in the target depth (at least 5 cm pressure depth) by 55% (95% CI 29 to 82%, *p* < 0.01) and in the duration of the release phase by 75 ms (95% CI 48 to 101 ms, p < 0.01). No significant difference was found regarding hands-off times, compression frequency or exhaustion.

**Conclusion:**

Physical strain during a realistic alpine rescue mission scenario at high altitude led to a significant reduction in quality of resuscitation. Resuscitation guidelines developed at sea level are not directly applicable in the mountain terrain.

## Background

High quality cardiopulmonary resuscitation (CPR) is a key factor in survival with good overall quality of life after out-of-hospital cardiac arrest. Recent guidelines on resuscitation put an increasing level of emphasis on high quality chest compressions. Only adequate chest compressions assure sufficient cerebral blood flow, and allow for good neurologic outcome [1].

Current evidence, especially on rate and frequency of chest compressions, target depth, and rate of change between those performing chest compressions is predominantly based on studies conducted at low altitude (< 1000 m above sea level) ( [2–4]. McDonald et al. (2013) [5] investigated the proportion of correctly performed chest compressions in an urban environment. These correctly performed chest compressions decreased from 52% after one minute to 39% after five minutes. The authors therefore recommended a change of helpers at least every two minutes. This was subsequently included in the ERC-guidelines of 2015 [1].

Recent years have shown a soaring development of mountain tourism, including a large share of elderly tourists and those with increased cardiovascular risk [6]. Mountain rescue services face increasing numbers of non-traumatic cardiac arrest calls [7, 8]. Although helicopter emergency medical service (HEMS) is widely established in the mountain terrain of developed countries, its use is not always possible due to visibility or weather conditions. This means that rescuers must often perform a fast, straining ascent to the patient, and then perform CPR on scene. As patients often deteriorate only during the helpers’ ascent, and exact time of cardiac arrest is not known, CPR usually cannot be withheld due to futility.

To date only few studies have addressed the issue of high-altitude exposure, leading to hypobaric hypoxia, and especially physical strain in these conditions, on quality of CPR.

Wang et al. [9] found a significant deterioration of quality of chest compressions at high altitude (3100 m). Participants were however flown to the mountain before performing CPR. Hence, physical strain before CPR was not studied, and the study does not reflect the situation of a wearisome ascent prior to resuscitation, often encountered as outlined above.

Narahara et al. [10] evaluated physiological response to CPR at high altitude (3700 m), and found significantly greater physical effort compared to the strain at sea level. They did, however, not measure the quality of CPR. In addition, rescuers were untrained office workers without any mountaineering experience, rested for 30 min after ascent and before beginning CPR, and performed chest compressions for only five minutes. This might not reflect real practice.

The aim of this work was to investigate the effects of physical strain on quality of cardiopulmonary resuscitation in a realistic high-altitude alpine rescue scenario.

## Methods

This was a randomised cross-over experiment (A-B-A sequence design) [11] in an alpine environment in Austria. The study was approved by the institutional review board of the Medical University of Vienna.

### Study subjects

A total of 20 mountain-experienced members of the Austrian Mountain Rescue Service with regular training in Basic Life Support (BLS) according to ERC guidelines at least 18 years of age were included.

### Study setting

The study was performed at low altitude (Lienz, East Tyrol, Austria, 673 m above sea level), and after a quick ascent from a mid-height base camp over 1213 m to a mountain shelter at 3454 m (Erzherzog Johann Hütte, Großglockner mountain).

### Intervention and measurement

After informed consent, subjects completed a standardized fitness questionnaire (FFB mot) [12]. The FFB is a validated test for overall fitness and divided into a total of 4 subgroups of 7 items each (strength, endurance, agility and coordination). Immediately before CPR, subjects performed testing using the modified nine-hole peg test (NHPT) [13]. This test of dexterity has already been used before as a measure of exhaustion after performing CPR [14–17]. Vital signs (HR, blood pressure, and SpO2) were measured as well.

Participants now performed BLS (CPR 30:2 with bag mask ventilation) on a manikin (Resusci Anne, Laerdal, Norway) for 16 min in groups of two, switching roles every two minutes. During CPR, quality of CPR (compression depth, frequency of compressions, portion of compression in the target area (at least 5 cm pressure depth), absolute distance of compressions (= pressure x frequency), hands-off times, duration of the release phase, and the percentage of time, in which chest compressions were performed) were continuously measured using a feedback device (M-Series, ZOLL-Chelmsford, MA). Subjects’ heart rate was also continuously measured during CPR using a chest strap (H7, Vantage V, Polar Electro Oy, Finland). Every two minutes, subjects were asked about their subjective exercise intensity, measured using the BORG-CR 10 [18].

After the scenario, vital signs and NHPT were measured again.

On the next day, participants performed a quick ascent from base camp to the mountain shelter using all their mission equipment and backpack, as they would in a real situation. Immediately after arrival at the mountain shelter, NHPT and vital signs were measured, and participants performed CPR. All measurements took place exactly as at baseline.

After return to baseline height at Lienz on a moderate pace and a resting period of one hour, participants performed CPR for a third time, again exactly as done before, including all measurements.

During all three scenarios, group assignment remained the same.

### Analysis

Sample size calculations were based on the primary outcome mean relative compression depth. In order to detect a difference of 5 mm with a standard deviation (SD) of 3.5 mm at a significance level of 5% at a power of 90%, a total of 16 subjects were needed. To allow for the study design, we decided to include a total of 20 participants in ten teams.

Continuous data are presented as mean ± standard deviation, count data are presented as absolute number and relative frequency. All outcome variables were compared between the “high altitude” and “baseline altitude” measurement. The assignment of the analysis to use baseline altitude data from before (i.e. “baseline altitude - high altitude” sequence) or after (i.e. “high altitude – baseline altitude” sequence) the high-altitude measurement was randomly chosen to take into account any potential learning effects. The remainder baseline measurements were discarded, which results in a randomised cross-over experiment.

The differences between high altitude and baseline and their 95% confidence intervals were calculated using linear random effects models to allow for the study design with ‘team’ as the unit of analysis and altitude as the covariable for each outcome separately. The null hypothesis of no difference between the altitudes was formally tested using the Wald test from the above models. We tested for an interaction of the differences with ascension speed. We performed sensitivity analyses to investigate properly randomised group allocation by comparing before-after with after-before effects. We also used the individual level analysis as a sensitivity analysis, knowing that hands-off time is more reliable at the team-level, and team-level analysis better reflects patient care. For data management and analysis, we used MS Excel and Stata (StataCorp, College Station, TX). A two-sided *p* value less 0.05 was generally considered significant.

## Results

### Characteristics of study subjects

A total of 20 subjects (three, 15% female) were included in the study. Body mass index (BMI) and FFB Mot showed high fitness levels (see Table 1 for baseline information). All subjects were able to complete the scenarios as planned.

**Table 1:** Demographics of study subjects

	Total (***n*** = 20)
**Age (years)** (mean, SD)	38 (12)
**Height (cm)** (mean, SD)	178 (6)
**Weight (kg)** (mean, SD)	74 (8)
**BMI (kg/m**^**2**^**)** (mean, SD)	23 (2)
**Smoker** (%)	2 (10%)
**FFB Mot** (mean, SD), max. 5	
Strength	5 (0)
Endurance	5 (1)
Agility	5 (0)
Coordination	5 (0)
**Backpack weight (kg)** (median, IQR)	10 (3)
**Ascent time (minutes)** (min to max, mean, SD)	104 to 246, 178 (40)

*BMI* Body mass index

### Main results

**Fig. 1:**
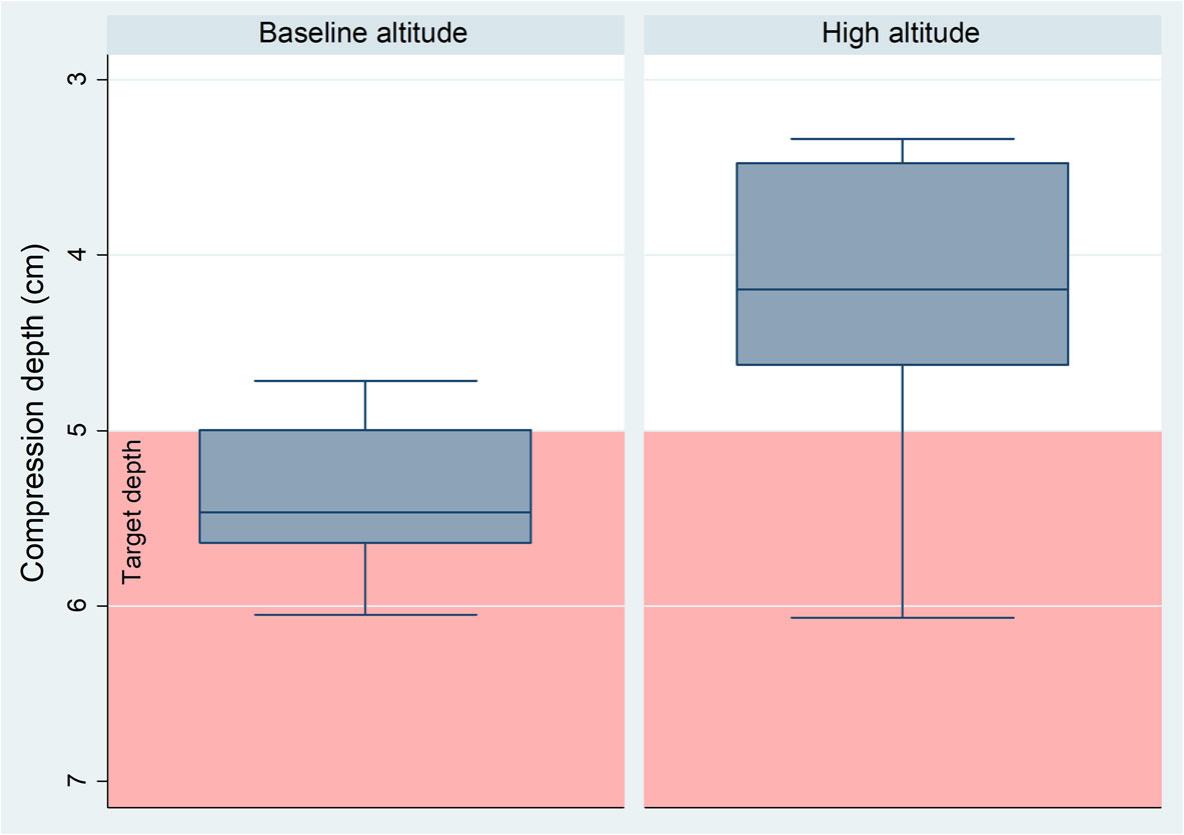
Mean compression depth at baseline and high altitude

The primary outcome “mean depth of chest compression” decreased significantly at high altitude compared to baseline by 1.0 cm (95% CI 0.5 to 1.3, *p* < 0.01) (See Fig. 1).

There was also a significant reduction in the percentage of compressions in the target area (at least 5 cm pressure depth) by 55% (95% CI 29 to 82%, *p* < 0.01) from 72% of all compressions to only 17%, and in the duration of the release phase by 75 ms (95% CI 48 to 101 ms, p < 0.01).

There was no significant difference in hands-off times, compression frequency, and proportion of time performing chest compressions. See Table 2 for details.

**Table 2:** Main results

	Baseline	High altitude	Difference (with 95% CI)	***p***-value
Compression depth (cm, mean, SD)	5.4 (1.6)	4.3 (0.9)	−1.0 (−1.3 to −0.5)	< 0.001
Compression frequency (pm, mean, SD)	120 (37)	123 (11)	3 (−4 to 10)	0.36
Compressions in target depth (%, mean, SD)	72 (31)	17 (34)	−55 (−82 to −29)	< 0.001
Hands-off time (s, mean, SD)	201 (63)	189 (20)	−13 (−27 to 2)	0.09
Duration of release phase (ms, mean, SD)	414 (132)	339 (82)	−75 (−101 to − 48)	< 0.001
Proportion of time performing chest compressions (%, mean, SD)	79 (23)	80 (2)	1 (− 6 to 3)	0.20

Regarding vital signs, range of heart rate increased at high altitude compared to baseline (126 before to 121 bpm after vs. 74 before to 84 bpm after CPR), and SpO2 decreased at higher altitude (87 to 88% vs. 97 to 98%). BORG scale as a subjective measure of exhaustion after 2 min of CPR was significantly higher at high altitude than at baseline (2.4 vs. 1.8); however this difference disappeared at the end of CPR (2.6 vs. 2.7). No differences were found in the NHPT as an objective measure of exhaustion between altitudes, neither before nor after CPR. See Table 3 for details.

**Table 3:** Vital signs and measures of physical strain of study subjects. All values are mean (SD)

	Baseline	High Altitude
**Systolic blood pressure (mmHg)**		
*Pre-CPR*	134 (10)	136 (14)
*Post-CPR*	139 (13)	129 (9)
**Diastolic blood pressure (mmHg)**		
*Pre-CPR*	78 (10)	83 (9)
*Post-CPR*	81 (11)	81 (8)
**Heart rate (beats/min)**		
*Pre-CPR*	74 (17)	126 (13)
*Post-CPR*	84 (16)	121 (18)
**SpO2 (%)**		
*Pre-CPR*	97 (2)	87 (3)
*Post-CPR*	98 (1)	88 (4)
**BORG - Scale**		
*After 2 min*	1.8 (1.0)	2.4 (0.8)
*End of CPR*	2.7 (1.1)	2.6 (0.8)
**NHPT (sec.)**		
*Pre-CPR*	24 (4)	26 (5)
*Post-CPR*	24 (4)	27 (6)

*NHPT* nine hole peg test

There was no interaction of ascension speed with the main effects. Sensitivity analysis showed no differences in per-group as compared to per-individual analysis. We also found no differences between those randomised to baseline-before as compared to those baseline-after, indicating successful randomisation and rejecting a learning effect as alternative explanation for the main effect.

## Discussion

Performing high-quality CPR requires a high level of physical effort even at sea level [5].

In a group of trained and experienced subjects, the physical strain of a realistic alpine rescue scenario at high altitude resulted in a significant reduction in quality of CPR.

To our knowledge this was the first study on the influence of physical strain at high altitude on quality of CPR. Our findings however complement previous studies, in which high-altitude CPR (without prior physical strain) was investigated. Wang and colleagues reported a mean compression depth of 4.9 cm at 3100 m as compared to 5.3 cm at sea level. This difference of 0.4 cm was distinctly less pronounced than our findings of a reduction of 1.0 cm. We believe this may be explained by the different scenario used in the study by Wang: Subjects were flown to the mountain and performed 5 min of chest compression-only CPR vs. a quick ascent from base camp to the mountain shelter, and 16 min of 30:2 CPR in groups of two [9]. Although this represents one common scenario, use of HEMS might often not be possible due to weather or daytime restrictions, as outlined before.

We found no difference in compression frequency, hands-off time, and proportion of time performing chest compressions. No previous data exists on the latter two, but regarding compression frequency, our findings are consistent with those of Wang et al. [9].

Our participants were members of the mountain rescue service with excellent physical fitness. This was reflected by only the slightest change of heart rate after performing CPR, both at low and high altitudes, and the low degree of physical exhaustion, both subjective (as measured by the BORG scale), and objective (as measured by the NHPT).

Although participants reported slightly higher BORG scales after 2 min of CPR at high altitude compared to low altitude, this difference disappeared over the full duration of 16 min. These findings are again distinctly different to previous reports. Wang et al., who studied health care professionals, who were however not professional mountaineers, found a severe increase in heart rate (81 before, 103 after CPR at sea level; 94 before, 115 after at high altitude, compared to 74 before and 84 after, and 126 before and 121 after in our study), as well as in BORG scale (6.7 at beginning, 13.8 at end on sea level; 9.6 at beginning, 14.4 at end on high altitude; 1.8, 2.7 and 2.4 and 2.6 in our study) [9]. Narahara reported similar findings in laymen, with BORG scales of 13 after CPR at sea level and 15 after CPR at 3700 [10]. Interestingly, despite these large discrepancies in physical exhaustion between professional mountain rescue personnel and other rescuers, the degree of hypoxemia in our participants (SpO2 88% at high altitude) was very similar to those in the studies of Wang (88%) and Narahara (88% at 2500 m; 80% at 3700 m).

Notably, despite the low degree of physical exhaustion reported by participants, quality of CPR was significantly worse at high altitude. This suggests that professional mountain rescue personnel were not able to judge the quality of their CPR at high altitude.

Our findings have severe implications on the performance of CPR in mountain terrain. Although the 2015 ERC guidelines for the first time encompassed distinct chapters on specific mountain emergencies, such as avalanches or hypothermia, the universal CPR algorithm (for non-hypothermic patients) still remains the same. This algorithm is based on findings from studies on low altitude and cannot be directly transferred to the environment of mountain rescue service.

We found that CPR performed by physically fit and highly trained professionals according to current BLS guidelines at high altitude led to a proportion of sufficient chest compressions of only 17%. This may seriously impair patient outcome. Future research should therefore focus specifically on different strategies to optimise quality of chest compressions at high altitude. These strategies could include more frequent change of roles (which, on the other hand, could increase hands-off times), use of mechanical CPR devices and feedback-devices (which, however, is limited by the weight rescuers could carry).

## Conclusion

Physical strain during a realistic alpine rescue mission scenario at high altitude led to a significant reduction in quality of resuscitation. Resuscitation guidelines developed at sea level are not directly applicable in the mountain terrain.

## Data Availability

The datasets used and/or analysed during the current study are available from the corresponding author on reasonable request.

## Abbreviations

95% CI: 95% confidence interval
BLS: Basic Life Support
BMI: Body mass index
CPR: Cardiopulmonary resuscitation
FFB: Fitness questionnaire
HEMS: Helicopter emergency medical service
HR: Heart rate
NHPT: Modified nine-hole peg test
SD: Standard deviation
SpO2: Oxygen saturation
